# Herbal Products and Their Active Constituents Used Alone and in Combination with Antifungal Drugs against Drug-Resistant *Candida* sp.

**DOI:** 10.3390/antibiotics10060655

**Published:** 2021-05-31

**Authors:** Anna Herman, Andrzej Przemysław Herman

**Affiliations:** 1 Faculty of Health Sciences, Warsaw School of Engineering and Health, Bitwy Warszawskiej 1920 18 Street, 02-366 Warsaw, Poland; 2 Department of Genetic Engineering, The Kielanowski Institute of Animal Physiology and Nutrition, Polish Academy of Sciences, Instytucka 3 Street, 05-110 Jabłonna, Poland; a.herman@ifzz.pl

**Keywords:** herbalproducts, herbal active constituents, drug-resistant *Candida* sp., antifungal drug

## Abstract

Clinical isolates of *Candida* yeast are the most common cause of opportunistic fungal infections resistant to certain antifungal drugs. Therefore, it is necessary to detect more effective antifungal agents that would be successful in overcoming such infections. Among them are some herbal products and their active constituents.The purpose of this review is to summarize the current state of knowledge onherbal products and their active constituents havingantifungal activity against drug-resistant *Candida* sp. used alone and in combination with antifungal drugs.The possible mechanisms of their action on drug-resistant *Candida* sp. including (1) inhibition of budding yeast transformation into hyphae; (2) inhibition of biofilm formation; (3) inhibition of cell wall or cytoplasmic membrane biosynthesis; (4) ROS production; and (5) over-expression of membrane transporters will be also described.

## 1. Introduction

In recent years, multidrug-resistant pathogens have become a serious health problem worldwide. Improper and extensive usage of antibiotics results in selective pressure supporting the rise of antibiotic-resistant microbes. At present, clinical isolates of *Candida* yeastare considered to be one of the highly resistant fungi to most commercially known antifungal drugs [1]. *Candida* is an opportunistic pathogen that can cause local and systemic infections in predisposed individuals, commonly affecting immunologically compromised patients and those undergoing prolonged antifungal drug treatment [2]. The most frequently clinicallyisolated drug-resistant species are *C. albicans*, *C. tropicalis*, *C. krusei*, *C. parasilopsis*, and *C. glabrata* [3]. To main synthetic drugs used against *Candida* sp. belongazoles (fluconazole, itraconazole, voriconazole, ketoconazole), polyenes (amphotericin B, nystatin), echinocandins (caspofungin, micafungin, anidulafungin), and allylamine (terbinafine) [1,4]. Due to the wide usage of azole drugs and prolonged antifungal therapy, the number of azole-resistant yeast isolates is stillincreasing [5]. Moreover, resistance to fluconazole triggers cross-resistance to other azoles or pathogen shifts from *C. albicans* toless sensitive species such as *C. glabrata* and *C. krusei* [6]. *C. glabrata* is naturally about 8-fold more resistant to fluconazole than *C. albicans* and easily develops further fluconazole resistance in prolonged therapy with this drug [7,8]. Therefore, it is necessary to search for more effective antifungal agents that would successfully act against such fungi. Some herbal products and their active constituents can meet these requirements [9,10,11,12,13,14]. Numerous herbal products showed strong antifungal activityagainst many drug-resistant *Candida* sp. acting alone or synergistically with the antifungal drug [15,16]. This activity of herbal products may lead to new choices for the treatment of infectious diseases. Combinedtherapy (1) expands the antimicrobial spectrum and increases its efficiency; (2) prevents the emergence of resistant mutants; (3) reduces undesirable effects and minimizes toxicity; (4) exhibitsgreater antimicrobial activity than that would be expected from each antimicrobial agents individually;and (5) allows obtainingan adequate therapeutic effect with relatively small doses when compared with a synthetic medication [17]. Moreover, herbalantimicrobial agents with different mechanisms of action have been introduced as more successful strategies to treatinfections involving drug-resistantpathogens [18]. In this paper, the current knowledge on herbal products and their active constituents with antifungal activity against drug-resistant *Candida* sp. used alone and in combination with antifungal drugswassummarized based onseveral electronic databases and hand-searched references. Moreover, the mechanism of such herbal products’action will be also described.

## 2. Literature Search Strategy

The Scopus and Google Scholar databases were searched for articles published from 2011 to the present. Search terms included ‘herbal products against drug-resistant *Candida* sp.’, ‘herbal products in combination with antifungal drugagainst drug-resistant *Candida* sp.’,‘herbal products against fluconazole-resistant *Candida* sp.’, ‘active constituents from herbs against drug-resistant *Candida* sp.’, ‘active constituents in combination with antifungal drug against drug-resistant *Candida* sp.’and ‘active constituents from herbs against fluconazole-resistant *Candida* sp.’. References from reviews presentingherbal products and their active constituents against drug-resistant *Candida* sp. were searched for additional articles and case reports. A manual search was also conducted based on citations in the published literature.

### 2.1. Inclusion and Exclusion Criteria

Selection criteria excluded articles that examined antifungal activity of herbal products and their active constituentsagainst *Candida* sp.without determining their drug-resistance. In addition, publications in languages other than English were excluded.

### 2.2. Study Selection

**Figure 1:**
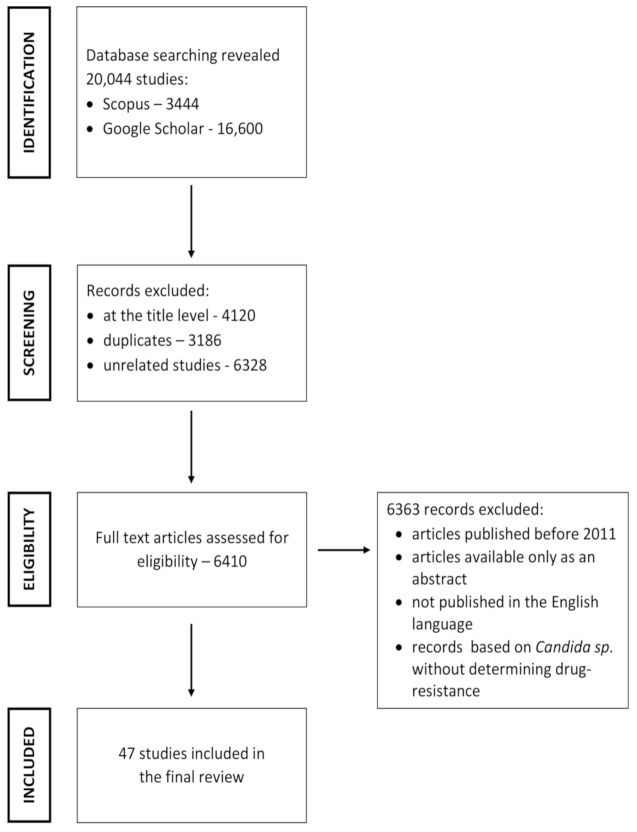
Search strategy used to identify relevant articles.

Overall, 20,044 articles were found in the databases. Of these, 13,634 articles were excluded at the title level, among them were alsoduplicates and unrelated articles. Furthermore, 6410 articles were excluded asnot meeting the inclusion criteria. Finally, 47 articles were usedfor the review (Figure 1).

## 3. Herbs Used against Drug-Resistant *Candida* sp.

Herbs have been used in traditional herbal medicine for many years. Herbal products may be also promising drugs against drug-resistant *Candida* sp. [9,10,11].

### 3.1. Herbal Products Used against Drug-Resistant Candida sp.

The aqueous extracts of *Lupinus various* seeds [19], *Echinophoraplatyloba* extract [20], hydroalcoholic extracts of *Andrographispaniculata* and *Achyranthesaspera* [21], as well as *Hibiscus sabdariffa* extract [22] strongly inhibited the growth of fluconazole-resistant *C. abicans*, while *Coriandrumsativum*, *Menthapiperita*, and *Punicagranatum* extracts showed superior antifungal activity against fluconazole-resistant *C. glabrata* [23]. Thecherrybark oak extract inhibited both fluconazole and nystatin-resistant *C. albicans* growth [24]. The ethanolic extract of *Azadiractaindica*, *Allium sativum*, *Cordiadichotoma*, *Ocimum sanctum*, *Syzygiumcumini*, and *Trigonellafoenumgrecum* were effective against all multidrug-resistant (fluconazole, clotrimazole, amphotericin B, itraconazole, ketoconazole, miconazole, and nystatin) *Candida* isolates (*C. albicans*, *C. tropicalis*, *C. krusei*, *C. glabrata*) [25]. Among them, the most effective against all *Candida* isolates was the ethanolic extract of *Allium sativum*.

### 3.2. Herbal Products and their Combination with Antifungal Drugs Used against Drug-Resistant Candida sp.

Unfortunately, data presenting theinteractions of herbs used in combination with conventional antifungal drugs against drug-resistant *Candida* strains is scacre. In some studies, it was shownthat *Ocoteaglomerata* extract [26], *Thymusbroussonetii* and *Thymus maroccanus* essential oils [27], *Thymus vulgaris* essential oil [28], *Citrusaurantium* essential oil [29] in combination with fluconazole; *Lavandulaangustifolia* essential oils [30], *Ocoteaglomerata* extract [26] in combination with ketoconazole; *Thymus maroccanus* and *Thymus broussonetii* essential oils [27], and *Citrusaurantium* essential oil [29] in combination with amphotericin B revealed a synergistic effect against clinical strains of the human pathogens such as *C. albicans*, *C. glabrata*, *C. tropicalis*,and *C. krusei*. However, it is not known whether these *Candida* strains weredrug-resistant or not. Extending the research on the interaction of herbs in combination with conventional antifungal drugs against drug-resistant *Candida* strains would enrich the scientific literature with new data.

## 4. Active Constituents Isolated from Herbs Used against Drug-Resistant *Candida* sp.

In recent years, an increasing number of studies was performed to discover new bioactive compounds of plant origin which may possibly control multidrug-resistant human pathogens [13]. Moreover, these active compoundscan display synergistic activity with antimicrobial drugs against many multidrug-resistant pathogens [12]. Some active constituents isolated from herbs can strongly inhibit drug-resistant *Candida* sp.as well as significantly enhance the anti-candidal activity of the drug in treating drug-resistant *Candida* sp.

### 4.1. Herbal Active Constituents Used against Drug-Resistant Candida sp.

Theberberineshowed antifungal activity against fluconazole-resistant *Candida* sp. (*C. albicans*, *C. tropicalis*, *C. parapsilosis*, *C. krusei*) [31].Thecurcumin–quercetin co-encapsulated in nanovesicles without hyaluronan had strong activity against fluconazole-resistant *Candida* isolates [32]. Among all tested monoterpenes (carvone, limonene, pinene, menthone, menthol, camphor, thujone, citronellol, piperitone), citronellol was the most potent compound with antifungal activity followed by α-pinene and menthol against fluconazole-resistant *Candida* sp. [33]. Pseudolaric acid B(PAB), a herbal-originated diterpene acid from *Pseudolarixkaempferi Gordon*, possesses inhibitory activity against fluconazole-resistant and fluconazole-susceptible strains of *C. tropicalis* [34].

### 4.2. Herbal Active Constituents and Their Combination with Antifungal Drugs Used against Drug-Resistant Candida sp.

It was also observedthat some active constituents isolated from herbs in combination with conventional antifungal drug showed synergistic antifungal activity against drug-resistant *Candida* strains. The gypenosides, the main active components of *Gynostemmapentaphyllum* in combination with fluconazole, revealed a synergistic antifungal activity against fluconazole-resistant *C. albicans* [35]. Highly active against fluconazole-resistant isolates and mature biofilm of *C. tropicalis* is were PAB in combination with fluconazole [34]. Eucalyptal D, a natural formyl-phloroglucinolmeroterpenoid, in combination with fluconazole significantly enhances the anticandidal activity of fluconazole in treating fluconazole-resistant *C. albicans* [36]. Alsogeraniol [37] and magnolol [38] enhanced the antifungal activity of fluconazole against fluconazole-resistant *C. albicans*. The *trans*-resveratrol and *cis*-resveratrol enhanced the azoles (ketoconazole, itraconazole) susceptibility on fluconazole-resistant *Candida* isolates [39].The combination of eugenol with fluconazole and azithromycin showed synergistic activity against pre-formed *C. albicans* and *Streptococcusmutans* mixed biofilms [40]. The most active phenol, carvacrol, and its combination with fluconazole, amphotericin B, nystatin, and caspofungin resulted insynergistic and additive effects against the resistant strain of *C. auris* and *C. albicans* [41]. The interaction between farnesol and fluconazole, itraconazole, voriconazole, posaconazole, and isavuconazole showed synergism against one-day-old *C. auris* biofilms [42]. Synergism and indifference were observed in the association of (R)-(+)-β-citronellol and amphotericin B, while the association between (S)-(−)-β-citronellol and amphotericin B displayed synergism, additivity, and indifference against strains of *C. albicans* and *C. tropicalis* [43].

## 5. Mechanism of Action of Herbal Therapy against Drug-Resistant *Candida* sp.

The mechanism of antifungal drug action against *Candida* sp. is onlypartially known [4]. It was found that antifungal drugstarget the biosynthesis of ergosterol, cell wall, and nucleic acid biosynthesis, leading to cell death [4,44]. Unfortunately, *Candida* sp. have developed many mechanismsby which they become resistant to the antifungal drugaction.In several antifungal-resistant *Candida* clinical isolates, the over-expression of membranes transporters (*Candida drug resistance* (*CDR1*, *CDR2*), *Candida multidrug resistance*— *CaMDR1*), altered ergosterol biosynthesis via mutation and/or over-expression of ergosterol pathway genes *ERG3* (encodingC-5 sterol desaturase; glycoprotein catalyzes the introduction of a C-5(6) double bond into episterol, a precursor in ergosterol biosynthesis), *ERG6* (encodingdelta(24)-sterol C-methyltransferase, which converts zymosterol to fecosterol by methylating C-24 in the ergosterol biosynthetic pathway), and *ERG11* (encodinglanosterol 14-alpha-demethylase, an enzyme in the cytochrome P450 family that catalyzes the C-14 demethylation of lanosterol to the form of 4,4’-dimethyl cholesta-8,14,24-triene-3-beta-ol, which is a step in ergosterol biosynthesis) as wellas altered sterol import were observed [4,44,45]. This all made these *Candida* clinical isolates resistant to some antifungal drugs.

**Figure 2:**
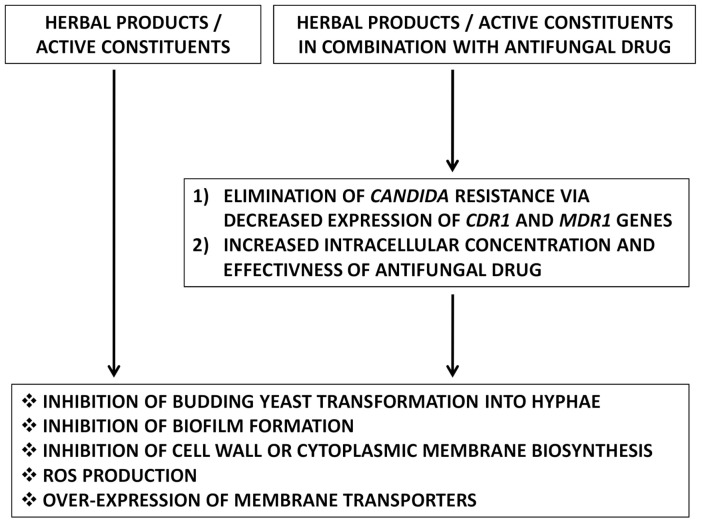
The mechanism of action of herbal products and their active constituents used alone and in combination with antifungal drug against drug-resistant *Candida* sp.

The mechanism of action of herbal products and their active constituents may be multi-directional (Figure 2). The possible mechanisms of herbs action on drug-resistant *Candida* sp.are based on (1) inhibition of budding yeast transformation into hyphae; (2) inhibition of biofilm formation; (3) inhibition of cell wall or cytoplasmic membrane biosynthesis; (4) reactive oxygen species (ROS) production; and (5) over-expression of membrane transporters [10,46]. Moreover, herbal products and their active constituents used alone act directly through the natural metabolic pathways to decreasedrug-resistant *Candida* sp.Whereas herbal products/active constituentsare used in combination with antifungal drug; in the first step, they decrease the drug resistance of yeast through the suppression of *CDR1* and multidrug resistant 1 (*MDR1*) gene expression.Then, they elevate the intracellular concentration of antifungal drugs and in turnincrease the effectiveness of those drugs against resistant *Candida* strains.

### 5.1. Inhibition of Budding Yeast Transformation into Hyphae

*C. albicans* grows as budding yeast which can transform into hyphae in response to various environmental or biological stimuli [47]. Yeast cell transformation to hyphaeis critical to the pathogenicity of *C. albicans*. Hyphae can attach to host cells, damage host tissue, and escape from host immune defenses [47]. Moreover, the hyphae form is responsible for the formation of multidrug-resistant biofilm. Some herbal products and their active constituents can inhibit yeast cell conversion to hyphae. *Pelargonium capitatum* and *Cymbopogon martini* essential oils can inhibit the major virulence factor of *C. albicans* as the germ tube formation [48]. Thegypenosides, the main active components of *Gynostemmapentaphyllum* in combination with fluconazole [35] and PAB, a herbal-originated diterpene acid from *Pseudolarixkaempferi Gordon* in combination with fluconazole [34] significantlyinhibit blastospore germination and early biofilm formation as well as the maturation of *C. albicans* and *C. tropicalis* biofilm. The (+)-Lyoniresinol-3α-*O*-β-d-glucopyranoside induced the accumulation of intracellular trehalose on *C. albicans* as a stress response to the drug, and it disrupted the dimorphic transition that forms hyphae [49]. Cinnamaldehyde showed impaired development of budding yeast cells to pseudo-hyphae and the absence of chlamydoconidia [50]. The selected flavones (luteolin, apigenin), flavonols (quercetin), and their glycosylated derivatives (quercitrin, isoquercitrin, rutin, and apigetrin) showed moderate activity in terms of reducing fungal hyphalgrowth [51]. Treatment of *C. albicans* cells with both apigetrin and its aglyconeapigeninlowers the number of cells growing in the hyphal form. Berberine hydrochloride (BBH) and fluconazole combination inhibited yeast adhesion, morphological hyphae transformation, and biofilm formation by downregulating the hyphal-specific genes *ALS3* (agglutinin-like protein 3)*, HWP1* (hyphal wall protein 1), and *ECE1* (extent of cell elongation protein 1) [52]. Moreover, this study also found that the vacuolar calcium 1 regulation genes (*YVC1*) and vacuolar calcium pump 1 gene (*PMC1*) are key targets for BBH and fluconazole combination, which increase cytoplasmic Ca^2+^ in resistant isolates, which might be critical for reversing biofilm-positive fluconazole-resistant *C. albicans* through yeast apoptosis induced by intracellular or mitochondrial high Ca^2+^ levels.

### 5.2. Inhibition of Biofilm Formation

The formation of biofilms makes treatment difficult and contributes to high rates of morbidity and mortality, thus representing one of the main virulence factors that contribute to the pathogenesis of candidiasis [53,54,55]. Therefore, it is crucial to explore alternative strategies to overcome the limitations of current therapies against *Candia* sp. infections associated with biofilms. An alternative to antifungal drugs used in the treatment of candidiasis can be herbal products and their active constituents with anti-biofilm potency. The *Ononisspinosa* effectively inhibited biofilms formed by *Candida* strains through inhibition of ergosterolsynthesis and leakage of cellular components [56]. Somered fruits (*Rubusidaeus*, *Vacciniummyrtillus*, *Vacciniummacrocarpon*, *Malpighiapunicifolia*) extracts showed a lack of antifungal activity but a significant anti-adhesion and anti-biofilm potency on *C. albicans* and *C. glabrata*,especiallythecranberry extract [57]. The *Hibiscus sabdariffa* extract was found to be significantly effective against fluconazole-resistant *C. albicans* isolated from patients but also to be a substitute for eradicating pre-formed biofilm and inhibiting the growth of *C. albicans* [22]. *Thymus kotschanus* essential oil exhibited anti-biofilm activity by a significant decrease of *als* gene expression, which leads to a decrease in the synthesis of ALS3—an important protein for fungal adhesion and biofilm formation [58]. In addition, ethanolic extract of *Boesenbergia rotunda* inhibitsthe biofilm formation of *C. albicans*, especially during the biofilm development stage, by reducing the cell surface hydrophobicity and suppressing the *ALS3* mRNA expression [59]. Furthermore, the active constituent isolated from *B. rotunda* had a stronger effect on *ALS3* mRNA expression (pinocembrin, pinostrobin) and significantly decreased the *ACT1* (actin 1) mRNA level (pinocembrin).The filamentous form decreased with pinocembrin rather than with pinostrobin and inhibitedthe stage of *C. albicans* biofilm development. The 6-shogaol extracted from ginger exhibited anti-biofilm activity by inhibiting biofilm formation and eradicating the preformed biofilms of *C. auris* [60]. The gypenosides, the main active components of *Gynostemmapentaphyllum*, in combination with fluconazole inhibit early biofilm formation, suppress drug efflux, and inhibit yeast–hyphalconversion [35]. The antifungal activities of purified plant metabolites (artemisinin and scopoletin) inhibited planktonic forms and pre-formed biofilms of *C. glabrata*, *C. guilliermondii*, and *C. parapsilosis* [61]. The isoquercitrin, apigetrin, and isoquercitrin exhibited an ability to act as biofilm formation inhibitors [51]. It was shown that protoberberines [62] and berberine [31] inhibitedbiofilm formation by *C. albicans*. Moreover, the combination of berberine and amphotericin B against *C. albicans*/*S. aureus* dual-species biofilms revealed that hyphalfilamentation of *C. albicans* and co-adhesion between *C. albicans*/*S. aureus* were considerably impaired by the treatment [63].

### 5.3. Inhibition of Cell Wall or Cytoplasmic Membrane Biosynthesis

The numerous herbal products and their active constituents target the biosynthesis of ergosterol, which is a unique cell membrane component, present only in fungi. The methanolic extract of *Ononisspinosa* [56] and *Coriarianepalensis* essential oil [64] effectively inhibited the biosynthesis of ergosterol, leading to disruption in the integrity of cell membrane and leakage of cellular components. Treatment of *C. albicans* with apigenin and rutin led to lower expression levels of ergosterol biosynthesis enzyme (*ERG11*), while apigenin and isoquercitrin up-regulated the expression of *ERG11*, since their application can lead to reduced susceptibility to azole antifungals [51]. Thekalopanaxsaponin A, a triterpenoidsaponin [65], and β-citronellol [66] decrease the ergosterol content of the cell membrane and contribute to the death of *C. albicans*, *C. glabrata*, and *C. tropicalis*. The cinnamaldehyde fungicidal mechanism of action is likely related to ergosterolcomplexation through binding to enzymes involved in the formation of the cytoplasmic membrane in yeast cells [50]. The fungicidal effect of *Coriandrumsativum* essential oil [67], protoberberines [62], and berberine [31] is a result of damage in the cell membrane and subsequent leakage of intracellular components such as DNA, which led to cell death of *Candida* sp., probably by apoptosis. The MCh-AMP1, a natural peptide from *Matricariachamomilla* L. flowers caused *C. albicans* cell death via increasing the cell membrane permeability by induced potassium leakage from the yeast cells [68]. Pseudolaric acid Bdestroys the cell integrity causing cell deformation, swelling, collapse, and outer membrane perforation [34]. The (R)-(+)-β-citronellol and (S)-(−)-β-citronelloldisplayed an effect on the fungal membrane but not on the fungal cell wallin *C. albicans* and *C. tropicalis* [43]. Moreover, anti-*Candida* activity through cell wall remodeling induction was observed after sodium houttuyfonate, berberine, palmatine, jatrorrhizine, cinnamaldehyde, and their combinations [69].

### 5.4. Reactive Oxygen Species (ROS)Production

The inhibition of cell wall or cytoplasmic membrane biosynthesis, cell wall remodeling, and disruption in the integrity of cell membrane leading to theleakage of cellular components outside the cell is one of the main mechanisms of action of herbal products and their constituents, but it is not the only one. For example, methanol extract of *Ocoteaglomerata* did not reveal effects on ergosterol biosynthesis; however, it led to an increase in intracellular ROS levels, decreased cell viability, and consequently, cell death [26]. Thekalopanaxsaponin A induced the accumulation of intracellular ROS, resulting in mitochondrial dysfunction as well a breakdown of the membrane barrier of *C. albicans*, causing the leakage of intracellular trehalose, the entrance of extracellular impermeable substance, and the decrease of ergosterol content, all of these contributed to the death of *C. albicans* cells [65]. The purified plant metabolites, artemisinin and scopoletin, were found to promote the accumulation of intracellular ROS by increasing oxidative stress on planktonic forms and pre-formed biofilms of *C. glabrata*, *C. guilliermondii*, and *C. parapsilosis* [61]. The MCh-AMP1, a natural peptide from *Matricariachamomilla* flowers, induced ROS production and caused cell death via increasing cell membrane permeability induced by potassium leakage from the *C. albicans* cells [68]. Thecarvacrol cause membrane disruption through inducedROS production and calcium dysfunction indicating by the Ca^2+^/calcineurinpathway [70]. Fu et al. [71] reported that the combination of baicalein and amphotericin B accelerated apoptosis accompanied by increased ROS and caspase activity viathe corresponding increase of gene CaMCA1 (*C. albicans* Metacaspase-1) in *C. albicans*.Theberberine serves as a potent ROS-inducing agent, disrupting the antioxidant system, especially in fluconazole-resistant *C. albicans* [72]. Interestingly, *C. albicans* exhibited efficient antioxidant response at lower concentrations but could not sufficiently alleviate berberine-induced oxidative stress occurring at concentrations greater than 250 μg/mL.

### 5.5. Over-Expression of Membrane Transporters

Literature data showed that a reduced level of intracellular antifungal drug accumulation in resistant *Candida* sp.correlates with the over-expression of the *CDR1* and *CDR2* genes encoding transporters of the ATP-binding cassette(ABC) family and the *CaMDR1*(*Candidaalbicans* Multi-Drug Resistance 1)gene coding a major facilitator superfamily (MFS) transporters [10,45,46]. Both types of pumps are known to cause drug-resistant *Candida* sp. [73]. The most common mechanism of fluconazole resistance in *C. albicans* is the failure of cells to accumulate the drug due to increased expression of the efflux proteins encoded by the *CDR1*, *CDR2*, and *MDR1* genes [46]. Some herbal products and their active constituentsdecreasethe expression of *CDR1* and *MDR1* genes and thereby inhibit the activity of these pumps, whichincrease the intracellular concentration of antifungal drugs (e.g.,fluconazole), thereby increasing the effectiveness of these drugs onthe resistant *Candida* strain. Keereedach et al. [74] notedthat Thai Cajuput essential oil from *Melaleucacajuputi* in combination with fluconazole inhibited the growth of fluconazole-resistant *C. albicans* clinical isolates by significant reduction of the *MDR1* gene expression level. The β-lapachone isolated from the lapacho tree reverted fluconazole resistance of *C. albicans* strains over-expressing transporters CaCdr2p (*C. albicans* drug-resistance protein 2, ATP-binding cassette transporter) and CaMdr1p (*Candida albicans* multidrug resistance protein 1, major facilitator superfamily transporter) by inhibiting these proteins activities [75]. The 6-shogaol extracted from ginger reduced the levels of aspartyl proteinases and downregulated the expression of the efflux pump-related *CDR1* gene in *C. auris* [60]. The antifungal capacity of selected flavones (luteolin, apigenin), flavonols (quercetin), and their glycosylated derivatives (quercitrin, isoquercitrin, rutin and apigetrin) on genes encoding efflux pumps (CDR1) were studied by Ivanov et al. [51].Apigenin and apigetrin exhibited the most prominent impact on lowering CDR1 levels, while the effect of other flavonoids was less profound. BBH and fluconazole combination inhibited intracellular fluconazole efflux due to key efflux pump gene *CDR1* downregulation, whereas fluconazole alone induced high *CDR1* transcription in resistant *C. albicans* strains [52]. BBH as the regulatorof drug transporter activity increases fluconazole sensitivity against *C. albicans*-resistant isolates. Eucalyptal D revealed the upregulation of *CDR1* and *CDR2* genes [36]. Eucalyptal D was speculated to be the substrate for Cdr1p and Cdr2p efflux pump and to competitively inhibit the excretion of fluconazole from fluconazole-resistant *C. albicans*. Furthermore, geraniol [37] and magnolol [38] were found as substrates for Cdr1p efflux pumps; they exert synergistic effects by the simultaneous upregulation of *CDR1* and *CDR2* expression as well as competitiveinhibition offluconazole efflux from fluconazole-resistant *C. albicans*.

## 6. Conclusions

This review suggests that the usage of herbs may be considered as important support for conventional antifungal therapy. Herbal products and their active constituents are reported to be potentially active against a wide variety of fungi including drug-resistant *Candida* sp. Moreover, the use of herbal products and their active constituents with antifungal drugs combination is likely to reduce the minimum effective dose of the drugs, thus minimizing their toxic side effects and the treatment cost. Unfortunately, little is known about the bioavailability of herbs and their active constituents. Most of the presented data arebased on in vitroexperiments carried out on drug-resistant *Candida* sp. isolated from skin, vagina, blood, pus swab, sputum, urine, gastric aspirate, or clinical isolates from a culture collection. Despite the fact that herbal compounds showed better antimicrobial activity against drug-resistant *Candida* strains when used in synergy with an antifungal drug, there are no preclinical and clinical in vivo studies confirming that these combinations can inhibit diseases caused by *Candida* yeasts. Therefore, more advancedin vivostudies are needed to fully evaluate this strategy of *Candida* treatment. However, the advancement in the techniques ofseparation, purification, and identification of bioactive compounds may enable obtaining new compoundsof plant origin which will be used as drugs for the treatmentofdrug-resistant *Candida* strains in the near future.

## Data Availability

Not applicable.
